# Neurodevelopmental justice: rethinking adolescent criminal responsibility in Puerto Rico

**DOI:** 10.3389/fpsyg.2025.1741945

**Published:** 2026-01-12

**Authors:** Edicer Ramírez-Rivera, Karen G. Martínez-González

**Affiliations:** Department of Psychiatry, University of Puerto Rico Medical Sciences Campus, San Juan, Puerto Rico

**Keywords:** adolescent brain development, criminal responsibility, forensic psychiatry, juvenile justice, Puerto Rico

## Abstract

Developmental neuroscience has increasingly informed debates about adolescent criminal responsibility by highlighting ongoing maturation of cognitive control, emotional regulation, and decision-making systems during adolescence. This Perspective examines how neuroscientific findings have been interpreted within legal and policy discussions of juvenile culpability, using Puerto Rico’s distinctive juvenile justice framework as a focal context. Rather than treating neurodevelopment as a causal explanation for criminal behavior, the article situates brain development within broader social, environmental, and legal conditions that shape adolescent conduct. Drawing on neuroscientific research, comparative legal analysis, and empirical evidence on justice-system outcomes, the article examines how concepts such as graduated responsibility, proportional accountability, and adolescents’ capacity for change align with Puerto Rico’s developmentally oriented juvenile justice model. Empirical evaluations of juvenile transfer policies are incorporated to assess public-safety, health, and developmental outcomes associated with adult-court prosecution versus juvenile-system processing. The discussion highlights both the promise and the limits of applying neuroscience to legal decision-making, emphasizing the importance of avoiding deterministic interpretations while recognizing adolescents’ heightened responsiveness to intervention and rehabilitation. The article argues that developmentally informed juvenile justice should prioritize fairness, contextualized responsibility, and access to trauma-informed, evidence-based supports. It also underscores the importance of interdisciplinary dialog and regionally grounded research for juvenile justice policy and practice in Puerto Rico.

## Introduction

Extreme acts of violence among youth raise enduring questions about crime, development, moral responsibility, and how justice systems should respond considering what neuroscience reveals about the adolescent brain (Welner et al., 2022; Borja-Martinez et al., 2024). In 2020, U.S. law enforcement agencies made approximately 424,300 arrests involving individuals under the age of 18, a 78% decline since 2006, but still representing a significant social and public health challenge [Office of Juvenile Justice and Delinquency Prevention (OJJDP), 2022]. While juvenile arrests have declined substantially, these cases still reveal deep questions about how society interprets adolescent conduct.

Neuroscience reveals that adolescence is not a period of fixed character or moral defect, but rather one of biological transformation. Functional and structural imaging indicate that the prefrontal cortex, responsible for planning and impulse control, undergoes protracted maturation, whereas the limbic system, which governs reward and emotional reactivity, matures earlier (Casey et al., 2008; Steinberg, 2009). This developmental maturity gap helps explain population-level tendencies for adolescents to understand rules yet have greater difficulty inhibiting impulses under conditions of stress or peer pressure (Cauffman and Steinberg, 2012).

Legal systems increasingly reflect these findings (Dünkel et al., 2020). Landmark U.S. Supreme Court cases such as Roper v. Simmons (2005), Graham v. Florida (2010), and Miller v. Alabama (2012) incorporated developmental evidence to restrict extreme sentencing and recognize the diminished culpability of adolescents (Bath et al., 2015; Fagan et al., 2016; Casey et al., 2022; Roper v. Simmons 2005; Graham v. Florida, 2010; Miller v. Alabama, 2012), as well as their increased vulnerability in other legal contexts such as custodial interrogation (J.D.B. v. North Carolina, 2011; Thompson and Fischer, 2015). Puerto Rico’s *Ley de Menores* (Minors Law) similarly adopts a rehabilitative ethos, positioning the island as a significant context for examining the intersection of neuroscience, law, and ethics (Gobierno de Puerto Rico, 1986, 2022; Da Nóbrega, 2024).

This article argues that rethinking juvenile justice through a developmental lens, sometimes referred to as neurodevelopmental justice, can humanize responses to youth crime by grounding accountability in evidence, empathy, and social context. Developmental science is not presented as a causal explanation for criminal behavior, but as a framework for assessing culpability, proportionality, and adolescents’ capacity for growth within legal decision-making.

## Adolescent neurodevelopment and culpability

The dual systems model posits an imbalance between an early-maturing socioemotional system and a later-developing cognitive control system (Steinberg, 2009). Heightened responsivity within affective and motivational systems has been associated with increased sensation-seeking, while ongoing maturation of prefrontal circuitry constrains the consistent regulation of emotions and goal-directed behavior, particularly in socially salient contexts (Casey et al., 2008; Gur, 2005, 2021; Liao, 2025). Behavioral research supports this distinction, showing that psychosocial maturity often lags behind cognitive reasoning, especially under conditions of emotional arousal or peer influence (Monahan et al., 2015).

Importantly, early neuroimaging work cautioned against interpreting these developmental patterns as categorical or abrupt. Casey and colleagues’ seminal review of structural and functional imaging studies emphasized that many aspects of prefrontal cortical organization reach near-adult levels by mid-adolescence, while other features, including connectivity and functional fine-tuning, continue to mature into later adolescence and early adulthood (Casey et al., 2005). This work highlighted substantial individual variability and underscored that developmental differences in neural maturation do not map directly onto behavioral outcomes. Such temporal dissociations help explain why peaks in adolescent risk-taking and offending often occur after many structural indices of cortical maturation have stabilized.

More recently, large-scale neuroimaging efforts have reinforced this gradualist interpretation. Using aggregated MRI data from over 100,000 individuals spanning infancy through late adulthood, normative brain charts demonstrate smooth, non-linear developmental trajectories with substantial overlap between adolescents and adults across cortical and subcortical measures, rather than discrete maturational stages (Bethlehem et al., 2022). These findings underscore the absence of a sharp neurobiological boundary separating adolescent and adult brains and caution against framing developmental differences as “fundamental.” Instead, they support a probabilistic understanding of developmental capacity that emphasizes gradients, overlap, and individual variability.

Diminished neurodevelopmental maturity therefore does not negate legal or moral responsibility; rather, it supports a graduated and context-sensitive understanding of responsibility (Casey et al., 2022; Scott et al., 2018). Adolescents generally possess moral understanding but may show reduced consistency in self-regulation, particularly in emotionally charged or socially demanding situations (Fontaine, 2008; Luna, 2012; Scott et al., 2018; Kramers-Olen, 2015). A commonly used heuristic in the literature describes adolescent development as involving earlier maturation of reward-sensitive systems relative to regulatory control networks (Steinberg, 2009; Casey et al., 2008; Tennison and Pustilnik, 2015), a formulation intended to capture population-level tendencies rather than deterministic mechanisms. Interdisciplinary scholarship at the intersection of neuroscience and law similarly cautions against treating neurodevelopmental evidence as dispositive of culpability, emphasizing instead its relevance for informing normative judgments about proportionality, capacity, and institutional response (Schleim, 2025; Tennison and Pustilnik, 2015).

When social adversity such as poverty, trauma, or substance exposure further strains developing regulatory capacities, the likelihood of impulsive or reactive behavior may increase for some youth (Evans-Chase, 2014; Popma et al., 2017). Recognizing this interplay does not absolve responsibility, but instead clarifies where legal responses, prevention strategies, and supportive interventions are most likely to be effective.

## Comparative statutory context: Puerto Rico and the continental United States

Puerto Rico’s *Ley de Menores* (Law 88-1986, amended by Law 47-2022) establishes juvenile court jurisdiction beginning at 13 years of age, limits automatic transfers to adult court, and allows juvenile oversight through age 21. The statute emphasizes mediation and diversion prior to prosecution, reflecting a developmentally oriented approach consistent with contemporary evidence on adolescent maturation (Gobierno de Puerto Rico, 1986, 2022; Pillay, 2019). The 2022 amendments reaffirmed chronological age as the primary jurisdictional criterion while introducing additional procedural protections within juvenile proceedings, including limitations on the use of restraints and certain institutional practices, and clarifying the statutory circumstances under which cases may be transferred out of juvenile court. Taken together, these provisions align with principles of proportional accountability supported by developmental research.

At the same time, this statutory scheme has become the focus of renewed legal and public debate, particularly regarding the criteria used to distinguish juvenile from adult court jurisdiction in serious offenses. Although jurisdiction is defined primarily by chronological age, these discussions have highlighted tensions between fixed legal thresholds and broader considerations of developmental capacity and psychosocial functioning (Howell et al., 2015). This ongoing discourse underscores the challenge of operationalizing developmental principles within legal systems that must balance statutory clarity, due process, and individualized assessment. Comparable tensions have been described in other jurisdictions, including the Netherlands, where adolescent criminal law has incorporated developmental evidence while explicitly cautioning against treating neuroscientific findings as determinative of individual culpability (Schleim, 2020).

This debate has intensified in recent policy discussions. In 2025, proposed legislative amendments would expand the categories of offenses for which minors could be prosecuted as adults and restrict the availability of mediation and diversion in certain contexts. These proposals have prompted public opposition from academic and professional organizations, which have argued that expanding adult-court exposure for youth risks undermining rehabilitative goals and conflicts with established developmental evidence (Tirado, 2025). Together, these developments illustrate an active policy debate over whether serious youth violence should be addressed through expanded adult-court jurisdiction or through strengthened juvenile-system mechanisms.

Empirical evaluations of juvenile transfer policies consistently indicate that prosecuting adolescents in adult court is associated with poorer public-safety, health, and developmental outcomes than retaining youth within juvenile systems. Systematic reviews and public-health task force reports have found that laws facilitating transfer to adult court are associated with higher rates of subsequent violence and recidivism, without evidence of a deterrent effect, including for serious and violent offenses (McGowan et al., 2007).

By contrast, juvenile-court processing is more frequently associated with reduced reoffending and improved long-term outcomes, particularly when youth have access to developmentally appropriate, evidence-based psychosocial interventions and coordinated health services (Sukhodolsky and Ruchkin, 2006; Owen et al., 2020). Longitudinal studies further indicate that exposure to adult correctional environments confers additional risk, including elevated rates of morbidity and premature mortality compared with youth processed exclusively within juvenile systems (Ruch et al., 2021; Silver et al., 2023; Nur and Silver, 2025; Sbeglia et al., 2024). Qualitative and mixed-method syntheses similarly underscore the importance of rehabilitative relationships, continuity of care, and age-appropriate services in promoting desistance and engagement among justice-involved youth (Moore et al., 2025). Taken together, evidence suggests that expanding adult-court exposure for adolescents may undermine both rehabilitative objectives and broader public-safety goals.

In comparative perspective, Puerto Rico’s juvenile justice system differs markedly from most continental U.S. jurisdictions. While Puerto Rico maintains mediation, diversion, and extended juvenile oversight through age 21, most U.S. states set the upper age of juvenile court jurisdiction at 17 and permit transfer to adult court through mechanisms such as judicial waiver or prosecutorial discretion [National Conference of State Legislatures (NCSL), 2023; Lee and Kraus, 2016; Quinn, 2002]. Although “raise-the-age” reforms have expanded juvenile jurisdiction in some states, none extend oversight to age 21. An overview of jurisdictional boundaries and justice orientations in Puerto Rico and the continental United States is presented in Table 1. This comparison illustrates how Puerto Rico’s extended juvenile jurisdiction reflects a distinctive application of developmental principles within a statutory framework. At the same time, it underscores the importance of consistent implementation across judicial and correctional contexts to ensure that developmentally informed provisions translate into practice.

**Table 1:** Juvenile-justice boundaries in Puerto Rico and the continental United States.

Dimension	Puerto Rico (Law 88-1986, amended by Law 47-2022)	Continental United States (typical practice)
Minimum age of criminal responsibility	13 years (article 4, amended 2022)	Typically 7–12 years, varies by state
Upper age of juvenile court jurisdiction	Up to 21 years for dispositions	Usually 17 years (16 in a few states)
Transfer to adult court	Limited to 15-year-olds charged with first-degree murder	Judicial waiver, prosecutorial discretion, or statutory transfer
System orientation	Rehabilitative and developmental; prioritizes mediation and diversion	Mixed and fragmented models; developmental science has informed some state-level reforms, but no uniform national standard

Comparison of statutory boundaries of juvenile-court jurisdiction and prosecutorial-transfer mechanisms between Puerto Rico and the continental United States. Puerto Rico maintains extended jurisdiction through age 21 and a predominantly rehabilitative orientation consistent with developmental science, whereas most U.S. jurisdictions limit juvenile oversight to age 17 and vary in transfer criteria [sources: Gobierno de Puerto Rico, 1986, 2022; National Conference of State Legislatures (NCSL), 2023].

## Environmental and psychosocial modulators of violence

The presence of adolescent neurodevelopmental characteristics alone cannot account for criminal behavior, as most adolescents do not engage in serious offending. Adolescent neurodevelopment occurs within complex social and environmental contexts. Chronic stressors such as trauma, poverty, and substance exposure interact with ongoing brain development, amplifying vulnerability in some youth while leaving others unaffected. Within the Puerto Rican context, 54.3% of minors under 18 lived below the federal poverty level in 2023, the highest rate among all U.S. jurisdictions (Family Data Center – Puerto Rico, 2024), underscoring the structural dimension of developmental risk. Early adversity disrupts prefrontal–limbic coordination, heightening vulnerability to reactive or aggressive behaviors (Evans-Chase, 2014; Williams, 2020; Corrêa et al., 2024).

Neuroimaging research also demonstrates atypical activation in fronto-amygdala and cerebellar circuits among juvenile offenders, reflecting dysregulation within networks that govern behavioral inhibition and emotion processing (Wang et al., 2025). Findings from Puerto Rico’s 2018–2020 *Consulta Juvenil* survey further indicate that approximately 4.8% of students in grades 7–12 reported illicit drug use within the previous year, highlighting the early onset of substance exposure during adolescence [Administración de Servicios de Salud Mental y Contra la Adicción (ASSMCA), 2020].

## Discussion and future directions

Across multiple disciplines, a growing body of evidence suggests that adolescents’ neural systems limit impulse control yet preserve an extraordinary capacity for learning and change (Steinberg, 2009; Scott et al., 2018). The same developmental immaturity that makes young people more prone to risk also makes them more responsive to guidance, support, and rehabilitation. When justice relies primarily on punishment, it risks deepening the very vulnerabilities that neuroscience helps us understand.

Developmental science reinforces long-standing criminological findings that adolescent offending cannot be separated from broader social and environmental conditions. Poverty, trauma, school failure, and exposure to violence shape neurodevelopment and moral reasoning long before any criminal act occurs. Viewing these behaviors in isolation from their social context reduces a public-health challenge to a moral defect. In contrast, a neurodevelopmental framework invites an approach to justice that prioritizes care, structure, and prevention.

Puerto Rico’s juvenile justice system reflects partial alignment with these principles through its rehabilitative orientation and age-appropriate jurisdictional limits. However, the practical realization of neurodevelopmental justice requires more than statutory language. It calls for investment in early prevention, access to mental-health care, and trauma-informed interventions within educational and correctional settings. Judges, prosecutors, and clinicians should be trained to interpret adolescent behavior through the lens of developmental neuroscience and psychosocial maturity (Cavanagh, 2022).

Regional research across the Caribbean and Latin America is also needed to strengthen this evidence base, as most current data derive from North American or European populations (Mercurio et al., 2020; Da Nóbrega, 2024). Expanding culturally contextualized studies would allow policymakers to craft responses that reflect local realities while maintaining global scientific standards.

Ultimately, neurodevelopmental justice is not an appeal for leniency but a call for fairness grounded in evidence. It recognizes that accountability and compassion are not mutually exclusive. Adolescents in conflict with the law need both clear boundaries and structured opportunities for growth, mentorship, and reintegration. A just system is measured not by the number of youths detained, but by how many regain the chance to mature into responsible, connected adults.
